# The predictive value of cardiovascular outcomes and mortality assessed by the C-reactive protein to albumin ratio in the UK Biobank

**DOI:** 10.1186/s12872-024-03995-9

**Published:** 2024-06-26

**Authors:** Per Wändell, Axel C Carlsson, Anders O Larsson, Johan Ärnlöv, Toralph Ruge, Andreas Rydell

**Affiliations:** 1 Division of Family Medicine and Primary Care, NVS Department, Karolinska Institutet, Alfred Nobels Allé 23, Huddinge, SE-141 83 Sweden; 2 Academic Primary Health Care Centre, Stockholm Region, Stockholm, Sweden; 3 Department of Medical Sciences, Uppsala University, Uppsala, Sweden; 4 Department of Emergency and Internal Medicine, Skånes University Hospital, Malmö, Sweden; 5 Department of Clinical Sciences Malmö, Department of Internal Medicine, Lund University, Skåne University Hospital, Malmö, Sweden; 6 School of Health and Welfare, Dalarna University, Falun, Sweden

**Keywords:** Cardiovascular mortality, Diabetes, Blood pressure, CRP, Albumin

## Abstract

**Background:**

The C-reactive protein/albumin ratio (CAR) seems to mirror disease severity and prognosis in several acute disorders particularly in elderly patients, yet less is known about if CAR is superior to C-reactive protein (CRP) in the general population.

**Methods:**

Prospective study design on the UK Biobank, where serum samples of CRP and Albumin were used. Cox regression analyses were conducted to assess all-cause and cardiovascular mortality, myocardial infarction, ischemic stroke, and heart failure over a follow-up period of approximately 12.5 years. The Cox model was adjusted for established cardiovascular disease (CVD) risk factors, including age, sex, smoking habits, physical activity level, BMI level, systolic blood pressure, LDL-cholesterol, statin treatment, diabetes, and previous CVD, with hazard ratios (HRs) and corresponding 95% confidence intervals (CIs). Analyses were also stratified by sex, CRP level (< 10 and ≥ 10 mg/ml) and age (< 60 and ≥ 60 years).

**Results:**

In total, 411,506 individuals (186,043 men and 225,463 women) were included. In comparisons between HRs for all adverse outcomes, the results were similar or identical for CAR and CRP. For example, both CAR and CRP, adjusted HRs for all-cause mortality were 1.13 (95% CI 1.12–1.14). Regarding CVD mortality, the adjusted HR for CAR was 1.14 (95% CI 1.12–1.15), while for CRP, it was 1.13 (95% CI 1.11–1.15).

**Conclusions:**

Within this study CAR was not superior to CRP in predictive ability of mortality or CVD disorders.

**Clinical trial registration number:**

Not applicable (cohort study).

## Introduction

The C-reactive protein/albumin ratio (CAR) have in several studies been shown to predict outcomes in acutely ill patients and particularly geriatric patients. Initially, CAR was proposed to predict mortality in acute medical ill patients [1]. However, an increased number of studies has shown the CAR to be predictive in several acute diseases. During the COVID-19 pandemic, CAR was shown to be predictive in acute ill geriatric patients [2], and CAR was able to predict hospital mortality among geriatric patients admitted to the emergency department (ED) [2, 3]. Other studies have found CAR to be of predictive value for outcomes in different malignant diseases as well as in infectious diseases specifically for geriatric patients [4–7]. Regarding CAR as a marker among healthy elderly, an earlier study including two Swedish cohorts, CAR was not consistently associated with cardiovascular and all-cause mortality [8]. Thus, CAR has shown to be of value for individuals with different disorder, but in the mentioned study not among healthy elderly. However, studying CAR in other and larger databases are warranted.

Regarding the two components in CAR, C-reactive protein (CRP) is an acute phase protein (APP) used as a marker of inflammation. CRP responses have been observed in various diseases, including malignancies of different types, acute inflammatory diseases, including acute infections and trauma. Additionally, these responses have proven to be predictive indicators of mortality. [9]. Moreover, elevated CRP levels have been found in several chronic diseases including diabetes, atherosclerosis in general, heart failure, stroke, and dementia as well as other neurogenerative disorders [10, 11]. Furthermore, CRP levels correlate with BMI, and the adipose tissue produces a large number of proinflammatory cytokines [12]. Interestingly, elevated CRP levels also seems to be associated with different stages of frailty [13].

Albumin serves as an acute phase reactant in addition to its fundamental physiological functions, which encompass the preservation of optimal osmotic colloidal pressure and facilitation of intravascular molecular transport. Moreover, it holds significance in the realms of lipid metabolism and thrombotic haemostasis. Low circulating albumin (hypoalbuminemia) has been associated with the severity of several diseases such as malignant diseases, chronic inflammatory diseases, diabetes mellitus acute diseases, sepsis [14] and also cardiovascular disease (CVD) [15]. Albumin is, in addition to its role to mirror states of inflammation, also used as a biomarker of malnutrition and poor health status [16], and is negatively associated with both frailty and sarcopenia [13].

Regarding CAR as a marker among healthy elderly, an earlier study including two Swedish cohorts, CAR was not consistently associated with cardiovascular and all-cause mortality [8].

Collectively, CAR appears to possess substantial predictive utility in assessing prognostic outcomes among acutely ill patients, although its application within the broader elderly population remains relatively understudied. Consequently, our primary objective in this investigation was to examine the association between CAR levels and the extended-term incidences of cardiovascular outcomes and all-cause mortality in the UK-biobank comprising about five hundred thousand community dwelling individuals. In line with prior research within this domain, we postulated that elevated CAR values would be linked to an augmented risk of adverse outcomes and mortality, also in comparison with CRP.

## Methods

### Study population

Between 2006 and 2010, the UK Biobank recruited approximately 500,000 individuals aged 40–69 years from the National Health Service register. Detailed information regarding the study design and methodology can be found elsewhere [17]. During the participants’ initial visit to the study centre, the research team conducted a comprehensive assessment, which included administering questionnaires, conducting brief interviews, performing physical tests, and collecting blood samples.

The blood collection vessels (vacutainers and collection pots) were processed using various automation systems to generate multiple aliquots for long-term storage. These aliquots are split equally: half stored in a fully automated − 80 °C working archive and the other half in a manual, nitrogen-vapour back-up archive at separate sites to prevent degradation from freeze-thaw cycles or loss due to archive site breakdown. Standard haematological tests were conducted on fresh whole blood within 24 h of collection for all participants. Conversely, the biochemistry assays, including albumin, were carried out after extraction from the freezer and thawing. However, only the tubes needed for the biochemistry assays were removed from the freezer and thawed, while the remaining aliquots on each plate were returned to the working archive still frozen, to prevent unnecessary freeze-thaw cycles.

**Fig. 1:**
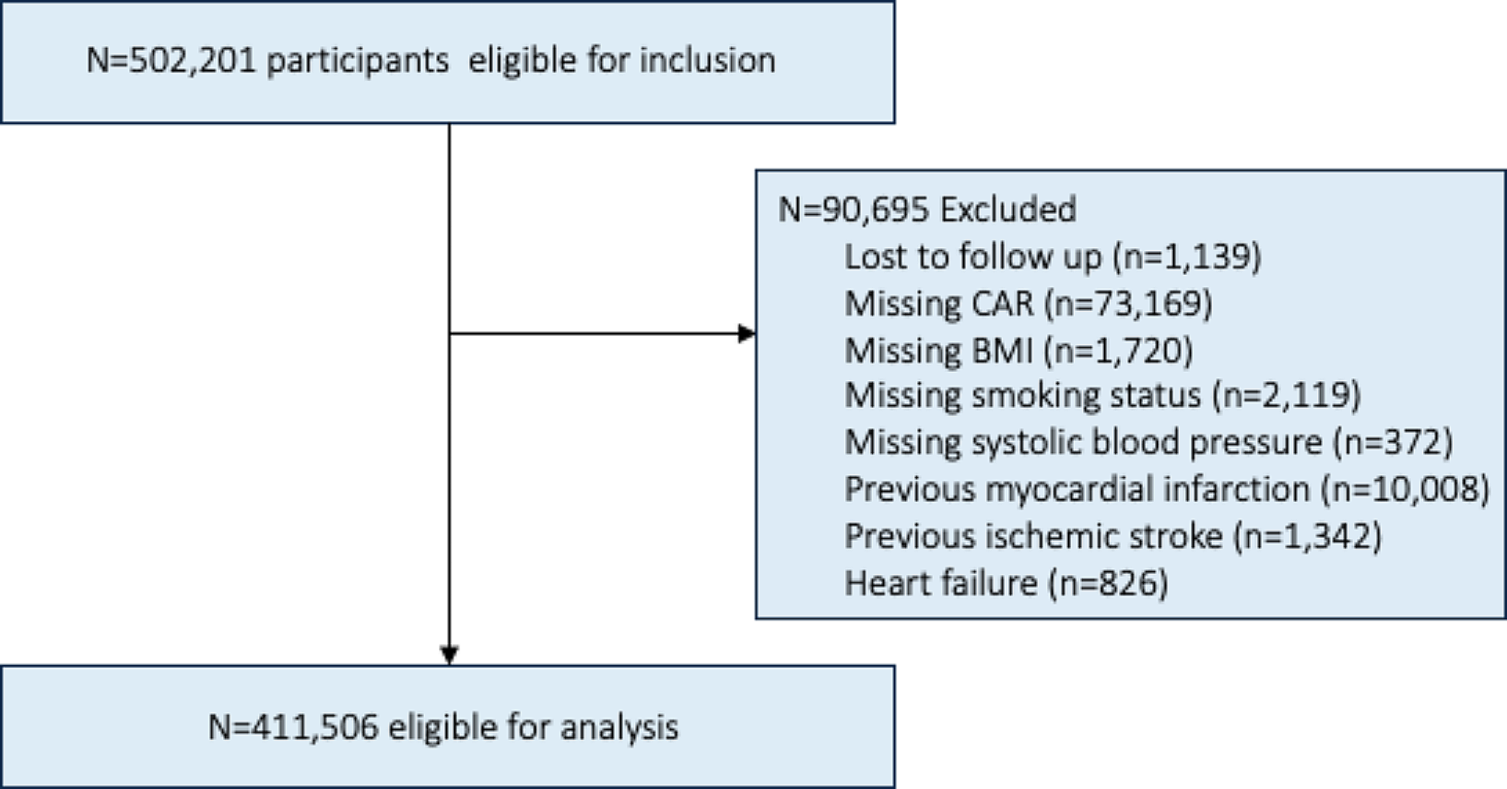
Flowchart showing the road to inclusion. BMI (Body Mass Index), CAR (C-reactive Protein/Albumin Ratio)

Individuals who withdrew their informed consent were consistently excluded from the UK Biobank, thus no count of their figures is available. Additionally, participants were excluded from this study if coded as lost to follow-up, had missing data on CAR, or lacked covariate information for body mass index (BMI), blood pressure, or smoking status. Furthermore, individuals who had experienced either a myocardial infarction (*n* = 10,008), ischemic stroke (*n* = 1,342), or had a diagnosis of heart failure (*n* = 826) were excluded (see Fig. 1). This study was carried out within the UK Biobank project 81508 and was approved by the Swedish Ethical Review Authority (2019–02328 and 2021-05762-02). Informed consent was obtained from all subjects and/or participants.

### Assessment of covariate data

Smoking status in the UK Biobank was assessed through a questionnaire^1^. In the present study, individuals were categorized as never smokers, former smokers, or current smokers. Individuals who chose the ‘prefer not to answer’ option regarding their smoking status were excluded. Pack years of smoking were determined using the formula: number of years smoked multiplied by the average number of cigarettes smoked per day divided by 20. In instances where pack years of smoking were not available, the mean value for the corresponding smoking status category and sex was utilized as a substitute (*n* = 54,045 for previous smokers and *n* = 9,118 for current smokers).

Physical activity levels in the UK Biobank were assessed using a questionnaire^2^. In the present study, individuals were categorized into one of three groups: sedentary, light, or high activity. This classification was based on the types of physical activities reported during the last four weeks. If data were missing, or if individuals were unsure, unwilling to respond, or only engaged in ‘walking for pleasure,’ they were classified as sedentary. Those participating in activities such as swimming, cycling, fitness exercises, bowling, or light/heavy do-it-yourself activities were categorized as having light physical activity. Individuals engaged in strenuous sports were classified as having a high level of physical activity.

Individuals were categorized as having diabetes at baseline (comprising both type 1 and type 2 diabetes) if they met any of the subsequent criteria:

1. Having an ICD-10 code ranging from E100 to E149 or an ICD-9 code ranging from 2500 to 2509 before the date of examination.
2. Exhibiting HbA1c levels equal to or exceeding 48 mmol/mol.
3. Receiving treatment involving insulin, metformin, pioglitazone, glipizide, glimepiride, rosiglitazone, or a combination of these medications during the examination.

Systolic blood pressure was assessed while individuals were in a seated position, with the measurement taken on the left arm (or right if more feasible). The arm was positioned at the level of the heart. Following a deliberate period of five slow breaths, blood pressure was measured using an Omron 705 IT electronic blood pressure monitor (OMRON Healthcare Europe B.V., Kruisweg 577, 2132 NA Hoofddorp). Blood pressure was measured twice. In this study, we utilized the average value of these two measurements, considering only the first if the second measurement was unavailable.

Low-density lipoprotein (LDL)-cholesterol levels were determined using enzymatic protective selection analysis (Beckman Coulter AU5800) with blood samples collected during the baseline examination. In individuals where LDL-cholesterol data were absent (*n* = 379 for women and *n* = 357 for men), the mean value for the corresponding sex within the overall study cohort was used as a replacement.

Information regarding statin treatment was gathered through self-reported medical conditions. Individuals were categorized as “yes” if they reported using any of the following statin medications: Rosuvastatin, Atorvastatin, Pravastatin, Simvastatin, Fluvastatin, Pitavastatin, and Lovastatin, or if the medication code or trade name corresponding to these statins was provided.

CRP levels were determined using high sensitivity immunoturbidimetric analysis on a Beckman Coulter AU5800. Albumin levels were measured through BCG analysis on the same instrument. CAR was calculated using the formula CRP/Albumin.

### Outcomes

The UK Biobank database encompasses detailed information on the occurrence and advancement of various diseases, alongside dependable data regarding mortality and causes of death. Its reliability and precision are fortified by seamless integration with national health registries, ensuring comprehensive coverage and minimizing the risk of loss to follow-up.

In this study, the definitions for cardiovascular mortality, myocardial infarction, ischemic stroke, and heart failure were based on specific diagnostic codes from the International Statistical Classification of Diseases and Related Health Problems, 10th Revision (ICD-10). Cardiovascular mortality was defined as individuals with a cause of death recorded with ICD-10 codes I00-I99. Myocardial infarction, including both non-ST-elevation myocardial infarction (NSTEMI) and ST-elevation myocardial infarction (STEMI), was recorded from inpatient hospital data. Additionally, individuals with ICD-10 codes I210-I214 and I219 were included in the definition of myocardial infarction. Ischemic stroke cases were identified using ICD-10 codes I630-I639 and I693. Heart failure cases were identified using ICD-10 codes I500-I509. The individuals were censored at the occurrence of the first event for each outcome, more specifically within the analysis pertaining to that particular outcome. If no event occurred, a predefined end date was utilized, which corresponded to the latest recorded occurrence of any adverse outcome within the dataset, i.e., November 12, 2021.

### Statistical analysis

A multivariable Cox proportional hazard model was utilized to investigate the relationship between CAR or CRP and the occurrence of five specific outcomes: overall mortality, cardiovascular mortality, myocardial infarction, ischemic stroke, and heart failure. The multivariable model incorporated a range of covariates, comprising age, sex, BMI, smoking status (never, former, and current), level of physical activity, systolic blood pressure, LDL cholesterol, statin treatment, baseline diabetes, and prior history of myocardial infarction, ischemic stroke, and heart failure. For the Cox analysis focusing on heart failure, individuals were excluded if they had a documented history of heart failure at baseline, given its chronic nature. Standardization was applied to both CAR and CRP due to their non-normal distribution, resulting in a mean value of zero. This approach ensures comparability and facilitates interpretation of the results. We finally analysed in individuals with high and low CRP using the cutoff of 10 mg/ml to see if any difference between CAR and CRP could be seen in individuals with low grade inflammation or more acute inflammation or infection could be detected [18].

Data management and statistical analyses were performed using Stata/SE 15.1 for Mac (StataCorp, TX, USA).

## Results

The study population and how it was obtained is shown in Fig. 1. In total, *n* = 411,506 individuals (186,043 men and 225,463 women) were included, and their baseline characteristics are shown in Table 1. Totally there were 28,349 all-cause deaths (16,256 men and 12,093 women), of which 5,207 were CVD deaths (3,469 men and 1,738 women). The average duration of follow-up for the adverse outcomes varied between 12.5 and 12.7 years.

**Table 1:** Basic values for study participants divided by sex, with mean values (standard deviations), or numbers (%) in groups for smoking habits and physical activity

Variable	Men (*n* = 186,043)		Women (*n* = 225,463)	All (*n* = 411,506)
Age years		56.5 (8.2)	56.3 (8.0)	56.4 (8.1)
Age < 60 years*		50.6 (5.6)	50.8 (5.6)	50.7 (5.6)
Age ≥ 60 years^ϕ^		64.2 (2.9)	64.0 (2.8)	64.1 (2.8)
BMI kg/m^2^		27.8 (4.2)	27.0 (5.1)	27.4 (4.7)
Smoking habits:				
Never		92,733 (48.8%)	134,814 (59.8%)	227,547 (55.3%)
Former		70,214 (37,7%)	70,682 (31.4%)	140,896 (34.2%)
Current		23,096 (12.4%)	19,967 (8.9%)	43,063 (10.5%)
Physical activity:				
Sedentary		10,836 (5.8%)	16,346 (7.3%)	27,182 (6.6%)
Light activity		172.686 (92.8)	208,368 (92.4%)	381,054 (92.6%)
Hard activity		2,521 (1.4%)	749 (0.3%)	3,270 (0.8%)
Systolic BP mm Hg		141 (17)	135 (19)	138 (19)
LDL-cholesterol mmol/L		3.5 (0.8)	3.6 (0.9)	3.6 (0.9)
Statin treatment		34,578 (18.6%)	24,845 (11.0%)	59,423 (14.4%)
Diabetes		22,352 (12.0%)	20,801 (9.2%)	43,153 (10.5%)
CAR		0.055 (0.100)	0.061 (0.102)	0.058 (0.101)
CRP mg/ml all		2.44 (4.29)	2.70 (4.32)	2.58 (4.31)
CRP < 10 mg/ml		1.80 (1.72)	1.98 (1.93)	1.90 (1.84)
CRP ≥ 10 mg/ml		19.56 (11.45)	17.85 (9.77)	18.53 (10.50)

Abbreviations: BMI (Body Mass Index), CVD (Cardiovascular disease), CAR (C-reactive Protein/Albumin Ratio), CRP (C-reactive Protein). *n = 236,233. ^ϕ^*n* = 175,273

For low CRP level, i.e., < 10 mg/ml, 394,735 individuals were included with 25,982 deaths, and CRP ≥ 10 mg/ml, 16,771 with 2,367; and for low age (< 60 years) 236,233 individuals with 8,386 deaths and high age (≥ 60 years) 175,273 individuals with 19,963 deaths.

In Tables 2, 3 and 4, HRs for CAR and CRP are presented, stratified by sex, and adjusted for age and CRP levels. HRs were comparable or identical between CAR and CRP for all outcomes, irrespective of subgroup level.

**Table 2:** The relative risk expressed as hazard ratios (HRs) and 95% confidence intervals (95% Cis) for different events among men (*n* = 186,043) for C-reactive protein/albumin ratio (CAR) and C-reactive protein (CRP), with adjustment for cardiovascular risk factors

Variable	All-cause mortality		CVD mortality		Myocardial infarction		Ischemic Stroke		Heart failure	
	HR	95% CI	HR	95% CI	HR	95% CI	HR	95% CI	HR	95% CI
Whole sample:										
CAR	1.14	1.13–1.15	1.15	1.13–1.17	1.07	1.06–1.09	1.07	1.04–1.09	1.10	1.09–1.12
CRP	1.14	1.13–1.15	1.15	1.13–1.17	1.07	1.06–1.09	1.07	1.04–1.09	1.10	1.09–1.12
Low CRP:										
CAR	1.55	1.49–1.61	1.72	1.60–1.85	1.30	1.24–1.37	1.32	1.23–1.43	1.48	1.40–1.57
CRP	1.50	1.45–1.56	1.66	1.54–1.79	1.28	1.22–1.35	1.29	1.20–1.39	1.45	1.37–1.53
High CRP:										
CAR	1.06	1.04–1.08	1.08	1.04–1.12	1.02	0.99–1.05	1.02	0.98–1.07	1.00	0.97–1.04
CRP	1.05	1.03–1.07	1.07	1.03–1.11	1.02	0.99–1.05	1.03	0.98–1.08	1.00	0.97–1.04
Low age:										
CAR	1.16	1.14–1.18	1.15	1.11–1.19	1.10	1.07–1.13	1.08	1.03–1.13	1.11	1.08–1.15
CRP	1.15	1.14–1.17	1.14	1.10–1.19	1.10	1.07–1.12	1.08	1.03–1.13	1.11	1.08–1.15
High age:										
CAR	1.13	1.12–1.14	1.15	1.12–1.17	1.06	1.04–1.08	1.06	1.04–1.09	1.10	1.08–1.12
CRP	1.13	1.12–1.14	1.15	1.12–1.17	1.06	1.04–1.08	1.06	1.04–1.09	1.10	1.08–1.12

All models were adjusted for: age, BMI, smoking status (never, former, and current), physical activity level, systolic blood pressure, LDL cholesterol, statin treatment, and diabetes at baseline. CRP divided into low CRP, < 10 mg/ml, and high CRP, ≥ 10 mg/ml; age divided into low age, < 60 years, and high age, ≥ 60 years

**Table 3:** The relative risk expressed as hazard ratios (HRs) and 95% confidence intervals (95% Cis) for different events among women (*n* = 225,463) for C-reactive protein/albumin ratio (CAR) and C-reactive protein (CRP) with adjustment for cardiovascular risk factors

Variable	All-cause mortality		CVD mortality		Myocardial infarction		Ischemic Stroke		Heart failure	
	HR	95% CI	HR	95% CI	HR	95% CI	HR	95% CI	HR	95% CI
Whole sample:										
CAR	1.13	1.12–1.14	1.10	1.07–1.14	1.10	1.07–1.12	1.07	1.05–1.10	1.12	1.10–1.14
CRP	1.13	1.11–1.14	1.10	1.07–1.14	1.09	1.07–1.12	1.07	1.04–1.10	1.12	1.10–1.14
Low CRP:										
CAR	1.35	1.30–1.41	1.30	1.17–1.46	1.34	1.25–1.44	1.24	1.14–1.35	1.37	1.28–1.47
CRP	1.33	1.28–1.39	1.27	1.14–1.41	1.31	1.22–1.40	1.21	1.11–1.32	1.35	1.26–1.44
High CRP:										
CAR	1.06	1.03–1.08	0.98	0.91–1.05	1.04	0.99–1.09	0.96	0.90–1.02	1.04	1.00-1.08
CRP	1.04	1.02–1.07	0.95	0.88–1.03	1.03	0.98–1.08	0.94	0.88-1.00	1.03	0.99–1.07
Low age:										
CAR	1.16	1.14–1.18	1.16	1.09–1.22	1.10	1.06–1.15	1.12	1.06–1.18	1.16	1.13–1.21
CRP	1.16	1.14–1.18	1.15	1.09–1.22	1.10	1.06–1.15	1.12	1.06–1.17	1.17	1.13–1.21
High age:										
CAR	1.12	1.10–1.13	1.09	1.05–1.13	1.09	1.06–1.12	1.05	1.02–1.09	1.11	1.08–1.13
CRP	1.11	1.10–1.13	1.08	1.04–1.12	1.09	1.06–1.12	1.05	1.01–1.08	1.11	1.08–1.13

All models were adjusted for: age, BMI, smoking status (never, former, and current), physical activity level, systolic blood pressure, LDL cholesterol, statin treatment, and diabetes at baseline. CRP divided into low CRP, < 10 mg/ml, and high CRP, ≥ 10 mg/ml; age divided into low age, < 60 years, and high age, ≥ 60 years

**Table 4:** The relative risk expressed as hazard ratios (HRs) and 95% confidence intervals (95% Cis) for different events among men and women together (*n* = 411,506) for C-reactive protein/albumin ratio (CAR) and C-reactive protein (CRP) with adjustment for cardiovascular risk factors

Variable	All-cause mortality		CVD mortality		Myocardial infarction		Ischemic Stroke		Heart failure	
	HR	95% CI	HR	95% CI	HR	95% CI	HR	95% CI	HR	95% CI
Whole sample:										
CAR	1.13	1.13–1.14	1.14	1.12–1.15	1.08	1.07–1.09	1.07	1.05–1.09	1.11	1.10–1.12
CRP	1.13	1.12–1.14	1.13	1.11–1.15	1.08	1.07–1.09	1.07	1.05–1.09	1.11	1.10–1.12
Low CRP:										
CAR	1.45	1.41–1.49	1.55	1.42–1.65	1.31	1.25–1.36	1.29	1.21–1.36	1.43	1.36–1.49
CRP	1.42	1.38–1.46	1.51	1.42–1.60	1.28	1.23–1.33	1.25	1.19–1.33	1.40	1.34–1.46
High CRP:										
CAR	1.06	1.04–1.07	1.05	1.02–1.08	1.03	1.00-1.05	1.00	0.96–1.04	1.02	1.00-1.05^ϕ^
CRP	1.05	1.03–1.06	1.03	1.00-1.06*	1.02	1.00-1.05^γ^	1.00	0.96–1.03	1.01	0.99–1.04
Low age:										
CAR	1.16	1.15–1.18	1.15	1.11–1.18	1.10	1.08–1.12	1.10	1.06–1.13	1.13	1.11–1.16
CRP	1.16	1.14–1.17	1.14	1.11–1.18	1.10	1.07–1.12	1.10	1.06–1.13	1.13	1.11–1.16
High age:										
CAR	1.12	1.11–1.13	1.13	1.11–1.15	1.07	1.05–1.09	1.06	1.04–1.08	1.10	1.09–1.12
CRP	1.12	1.11–1.13	1.13	1.10–1.15	1.07	1.05–1.09	1.06	1.04–1.08	1.10	1.09–1.12

All models were adjusted for: age, sex, BMI, smoking status (never, former, and current), physical activity level, systolic blood pressure, LDL cholesterol, statin treatment, and diabetes at baseline. *Denotes p-value 0.060, n.s. (95% CI 0.999–1.072). ^γ^Denotes p-value 0.092 (0.996–1.052). ^ϕ^Denotes p-value 0.096 (0.996–1.046). CRP divided into low CRP, < 10 mg/ml, and high CRP, ≥ 10 mg/ml; age divided into low age, < 60 years, and high age, ≥ 60 years. Abbreviations: BMI (Body Mass Index), CVD (Cardiovascular Disease), CAR (C-reactive Protein/Albumin Ratio), CRP (C-reactive Protein)

## Discussion

### Main findings

We obtained nearly identical results for CAR and CRP across most outcomes, with only minor differences observed among individuals with low CRP levels. Thus, the clinical significance of analysing the CAR in the general elderly population seems to be of limited value beyond CRP.

### Comparisons with other studies

Most earlier studies have analysed CAR in acute situations, such as malignant diseases of different kinds, acute inflammatory diseases also including acute infections, and trauma, where CAR have been shown to be predictive for mortality [9] The exposure of an acute illness including disorders such as an acute inflammation or infection, or a trauma, will induce an acute-phase reaction. This acute reaction will involve several different pathophysiological mechanisms, i.e., fever, leukocytosis, and a stimulation of the productions of APPs in the liver by cytokines. APP’s are regarded as negative and positive APPs, where negative APP’s include albumin and transferrin, and positive APP’s include CRP, thrombin, and d-dimer protein [19].

More detailed, CAR has been shown to be predictive of mortality among patients with many acute diseases. Yet, whether CAR was superior to CRP was not always the aim of the studies. Studies where CAR has been shown to be superior to CRP include some acute diseases, e.g. patients with different disorders at emergency departments (ED) [2], critically ill patients [20], and patients with sepsis [7], stroke [21], and myocardial infarction [22]. In other studies of acute diseases, CAR has been shown to be predictive, but not in direct comparison to CRP, e.g., patients with different disorders at emergency departments (ED [3], or respiratory diseases, also including acute COVID-19 infections [23], and pneumonia [24]. Furthermore, CAR has also been found to be prognostic in different chronic diseases, and superior to CRP in pulmonary arterial hypertension [25]. In other chronic diseases, CAR has shown to be predictive but not compared to CRP, including advanced malignant diseases [4–6, 26]. Finally, CAR has been suggested to reflect frailty or malnutrition among especially elderly patients, and thus not compared to CRP [27, 28].

### Mechanisms

In several studies CRP has been found to be strongly associated with mortality [9]. Our study thus adds to this of CRP as a general prognostic marker for mortality in the general population.

Regarding the other component of CAR, circulating albumin, it reflects both the inflammatory and the nutritional status. Among elderly cohorts, a study of 70-year-old community dwelling individuals, low concentrations of serum albumin were found to be associated with all-cause mortality [29], and of 80-year-old community dwelling individuals between low serum albumin and both all-cause and CVD mortality [30]. As regards the development for SIRS (Systemic Inflammatory Response Syndrome) in individuals in the general population after surgery, a significant association was found between increased CAR and development of postoperative SIRS [31]. In contrast to these findings, other studies, e.g. including patients with renal impairment, found no association between albumin level and CVD after adjustments for CRP [32–34], and no association between low serum albumin and CVD or cancer mortality was shown in a study of Japanese 70-year-old community-dwelling population although an association with total mortality was found [29]. Concluding the cited studies, the evidence indicates that the association between low serum albumin and specifically CVD comorbidity in the general population is rather weak.

Thus, by combining both CRP and albumin, CAR reflects both the inflammatory and the nutritional state of the patients and has been to be valuable in several both acute and chronic disorders, especially among elderly. However, in the studied included cohort individuals were probably too healthy and few were in a state of frailty or sarcopenia [13]. CAR probably could show a predictive value in cohorts including elderly and with higher frailty.

### Clinical implications

In this study of men and women from the general population, the predictive ability of CAR was found to be similar to that of CRP. Thus, in a healthy population the use of CAR does not seem to improve the prediction of mortality and CVD outcomes than CRP alone. As CAR has been found to be predictive in individuals with different acute and chronic diseases, it would be of importance to study frail individuals and populations.

### Strengths and limitations

The present study has several strengths. The cohort is rather large and includes a detailed characterization of the study population including many established risk factors.

Several noteworthy limitations are associated with the UK Biobank sample, including its limited ethnic diversity, reliance on self-reported data, susceptibility to selection bias, and the presence of a healthy volunteer effect.

## Conclusion

CAR showed no superiority in predicting neither mortality nor CVD disorders over CRP alone. Thus, our findings do not provide additional support for the use of CAR for mortality prediction in elderly in clinical practice in the general population.

## Data Availability

The UK biobank is accessible for researchers from all over the world, https://www.ukbiobank.ac.uk/.

## Footnotes

1 https://biobank.ndph.ox.ac.uk/showcase/refer.cgi?id=113241.

2 https://biobank.ndph.ox.ac.uk/showcase/refer.cgi?id=100475.

## References

[1] Fairclough E, Cairns E, Hamilton J, Kelly C (2009). Evaluation of a modified early warning system for acute medical admissions and comparison with C-reactive protein/albumin ratio as a predictor of patient outcome. Clin Med (Lond).

[2] Oh J, Kim SH, Park KN, Oh SH, Kim YM, Kim HJ, Youn CS (2017). High-sensitivity C-reactive protein/albumin ratio as a predictor of in-hospital mortality in older adults admitted to the emergency department. Clin Exp Emerg Med.

[3] Ayranci MK, Kucukceran K, Dundar ZD (2021). NLR and CRP to albumin ratio as a predictor of in-hospital mortality in the geriatric ED patients. Am J Emerg Med.

[4] Liang Y, Xiao W, Guan YX, Wang W, Chen HY, Fang C, Zhang X, Zhou ZW (2017). Prognostic value of the C-reactive protein/Albumin ratio (CAR) in patients with operable soft tissue sarcoma. Oncotarget.

[5] Liao CK, Yu YL, Lin YC, Hsu YJ, Chern YJ, Chiang JM, You JF (2021). Prognostic value of the C-reactive protein to albumin ratio in colorectal cancer: an updated systematic review and meta-analysis. World J Surg Oncol.

[6] Liu Y, Chen S, Zheng C, Ding M, Zhang L, Wang L, Xie M, Zhou J (2017). The prognostic value of the preoperative c-reactive protein/albumin ratio in ovarian cancer. BMC Cancer.

[7] Ranzani OT, Zampieri FG, Forte DN, Azevedo LC, Park M (2013). C-reactive protein/albumin ratio predicts 90-day mortality of septic patients. PLoS ONE.

[8] Wandell P, Carlsson AC, Larsson A, Arnlov J, Feldreich T, Ruge T. The C-reactive protein albumin ratio was not consistently associated with cardiovascular and all-cause mortality in two community-based cohorts of 70-year-olds. Scand J Clin Lab Invest 2023:1–5. 10.1080/00365513.2023.2255971 37702518

[9] Li Y, Zhong X, Cheng G, Zhao C, Zhang L, Hong Y, Wan Q, He R, Wang Z (2017). Hs-CRP and all-cause, cardiovascular, and cancer mortality risk: a meta-analysis. Atherosclerosis.

[10] Ho KM, Lipman J (2009). An update on C-reactive protein for intensivists. Anaesth Intensive Care.

[11] Luan YY, Yao YM (2018). The clinical significance and potential role of C-Reactive protein in chronic inflammatory and neurodegenerative diseases. Front Immunol.

[12] Larsson A, Hansson LO, Akerfeldt T (2013). Weight reduction is associated with decreased CRP levels. Clin Lab.

[13] Picca A, Coelho-Junior HJ, Calvani R, Marzetti E, Vetrano DL (2022). Biomarkers shared by frailty and sarcopenia in older adults: a systematic review and meta-analysis. Ageing Res Rev.

[14] Paliogiannis P, Mangoni AA, Cangemi M, Fois AG, Carru C, Zinellu A (2021). Serum albumin concentrations are associated with disease severity and outcomes in coronavirus 19 disease (COVID-19): a systematic review and meta-analysis. Clin Exp Med.

[15] Ronit A, Kirkegaard-Klitbo DM, Dohlmann TL, Lundgren J, Sabin CA, Phillips AN, Nordestgaard BG, Afzal S (2020). Plasma albumin and Incident Cardiovascular Disease: results from the CGPS and an updated Meta-analysis. Arterioscler Thromb Vasc Biol.

[16] Anderson CF, Wochos DN (1982). The utility of serum albumin values in the nutritional assessment of hospitalized patients. Mayo Clin Proc.

[17] Sudlow C, Gallacher J, Allen N, Beral V, Burton P, Danesh J, Downey P, Elliott P, Green J, Landray M (2015). UK biobank: an open access resource for identifying the causes of a wide range of complex diseases of middle and old age. PLoS Med.

[18] Ma Q, Zhou Y, Zhai G, Gao F, Zhang L, Wang J, Yang Q, Cheng W (2016). Meta-analysis comparing Rosuvastatin and Atorvastatin in reducing concentration of C-Reactive protein in patients with hyperlipidemia. Angiology.

[19] Jain S, Gautam V, Naseem S (2011). Acute-phase proteins: as diagnostic tool. J Pharm Bioallied Sci.

[20] Park JE, Chung KS, Song JH, Kim SY, Kim EY, Jung JY, Kang YA, Park MS, Kim YS, Chang J et al. The C-Reactive Protein/Albumin ratio as a predictor of Mortality in critically ill patients. J Clin Med 2018, 7(10). 10.3390/jcm7100333 PMC6210319 30297655

[21] Kocaturk M, Kocaturk O (2019). Assessment of relationship between C-reactive protein to albumin ratio and 90-day mortality in patients with acute ischaemic stroke. Neurol Neurochir Pol.

[22] Cinar T, Cagdas M, Rencuzogullari I, Karakoyun S, Karabag Y, Yesin M, Sadioglu Cagdas O, Tanboga HI (2019). Prognostic efficacy of C-reactive protein/albumin ratio in ST elevation myocardial infarction. Scand Cardiovasc J.

[23] Cordas Dos Santos DM, Liu L, Gerisch M, Hellmuth JC, von Bergwelt-Baildon M, Kunz WG, Theurich S. Risk stratification based on a pattern of immunometabolic host factors is Superior to Body Mass Index-based prediction of COVID-19-Associated respiratory failure. Nutrients 2022, 14(20). 10.3390/nu14204280 PMC9611334 36296963

[24] Kunutsor SK, Laukkanen JA (2022). Serum C-reactive protein-to-albumin ratio is a potential risk indicator for pneumonia: findings from a prospective cohort study. Respir Med.

[25] Cerik IB, Dindas F, Koyun E, Dereli S, Sahin A, Turgut OO, Gul I (2022). New prognostic markers in pulmonary arterial hypertension: CRP to albumin ratio and uric acid. Clin Biochem.

[26] Xu HJ, Ma Y, Deng F, Ju WB, Sun XY, Wang H (2017). The prognostic value of C-reactive protein/albumin ratio in human malignancies: an updated meta-analysis. Onco Targets Ther.

[27] Eraslan Doganay G, Cirik MO (2021). Determinants of prognosis in geriatric patients followed in respiratory ICU; either infection or malnutrition. Med (Baltim).

[28] Sanson G, Bertocchi L, Dal Bo E, Di Pasquale CL, Zanetti M (2018). Identifying reliable predictors of protein-energy malnutrition in hospitalized frail older adults: a prospective longitudinal study. Int J Nurs Stud.

[29] Takata Y, Ansai T, Yoshihara A, Miyazaki H (2012). Serum albumin (SA) levels and 10-year mortality in a community-dwelling 70-year-old population. Arch Gerontol Geriatr.

[30] Takata Y, Ansai T, Soh I, Awano S, Sonoki K, Akifusa S, Kagiyama S, Hamasaki T, Torisu T, Yoshida A (2010). Serum albumin levels as an independent predictor of 4-year mortality in a community-dwelling 80-year-old population. Aging Clin Exp Res.

[31] Chen C, Chen X, Chen J, Xing J, Hei Z, Zhang Q, Liu Z, Zhou S (2022). Association between Preoperative hs-crp/Albumin ratio and postoperative sirs in Elderly patients: a Retrospective Observational Cohort Study. J Nutr Health Aging.

[32] Stenvinkel P, Heimburger O, Paultre F, Diczfalusy U, Wang T, Berglund L, Jogestrand T (1999). Strong association between malnutrition, inflammation, and atherosclerosis in chronic renal failure. Kidney Int.

[33] Yeun JY, Levine RA, Mantadilok V, Kaysen GA (2000). C-Reactive protein predicts all-cause and cardiovascular mortality in hemodialysis patients. Am J Kidney Dis.

[34] Zimmermann J, Herrlinger S, Pruy A, Metzger T, Wanner C (1999). Inflammation enhances cardiovascular risk and mortality in hemodialysis patients. Kidney Int.

